# 

**Published:** 1892-03-05

**Authors:** 


### GENERAL CHARITIES.

[This Column is devoted to an account of work of charitable institutions other than Medical Charities. The Editor will be glad to receive reports or news of work at 140, Strand. *Philanthropy* should be plainly written on the envelope.]

A Yorkshire resident has offered anonymously, as "a friend to sailors," a mission steamer, valued at £300, for the use of the Mission to Seamen on the River Tees.

Under the will of the late Bishop Philpott a legacy of £10,000 will accrue on the death of his widow to the Church Pastoral Aid Society, and a similar sum to the Church Missionary Society. The late Mr. Charles Ravenhill, of the Stock Exchange, has bequeathed £500 to the Stock Exchange Benevolent Fund, and larger sums to several hospitals.

Donations to the amount of over £3,200 were announced at the recent anniversary dinner, in aid of the funds of the **LONDON ORPHAN ASYLUM**, Watford, at which Mr. Leopold de Rothschild presided. The Chairman spoke in high terms of the value of the Charity, which as he remarked, does everything which can be done for the education and amusement of its inmates. It is worthy of note that no death has occurred in the institution during the past four years. During the past year 112 orphans were admitted, and at the recent January election 40 more were elected. The work is not limited to any one class, but is extended to children from any part of the kingdom.

### SCRAPS AND GLEANINGS.

THE Southampton Hospital Sunday Fund for the past year amounted to £1,083 8s. 1d.

MR. GEORGE NEWNES, M.P., will preside at the annual festival of the Royal Hospital for Incurables, on May 18th, at the Albion.

BY a system of weekly payments the colliers in the Wigan District contribute annually nearly £4,000 to the Leigh and Wigan Infirmary.

THE Brompton Hospital for Consumption has received a donation of 100 guineas to name a memorial bed, also a gratuity of £545 9s. from the Hospital Saturday Fund.

GUY'S Hospital owns large estates in Herefordshire, Lincolnshire, and Essex, which in 1875 brought in a net revenue of £41,840. Last year the income had fallen to £27,580.

MR. E. A. PEARN, of Compton Leigh, near Plymouth, has offered to erect and endow a convalescent home at Compton Leigh, for the use of patients from the South Devon and the Royal Albert Hospitals.

THE Zenana Bible and Medical Mission, London, have lately received £50 from the Grocers, £50 from the Goldsmiths, £21 from the Mercers, £10 from the Clothworkers, and £10 10s. from the Skinners' Companies.

THE 24th anniversary dinner in aid of the funds of the French Hospital and Dispensary took place last Saturday evening at the Hotel Métropole, M. Waddington presiding. A list was read of the principal donations that had been received, amounting to £2,500.

THE Centenary Bazaar in aid of the Belfast Royal Hospital realized £3 573 14s. 4d. net after paying expenses, which includes £834 contributed specially to make life governors. This large amount exceeded the most sanguine expectations, and freed the hospital from debt.

THE annual report of the Oldham Infirmary shows that 4,577 patients were treated during the year. The income amounted to £3,866 7s. 10d., and the expenditure to £3,797 2s. 2d. The year commenced with an adverse balance £781 12s. 10d. which is now reduced to £711 17s. 2d.

THE sum of £1,000 (part of a munificent gift of £10,000 from Mr. Eli Lees to the above institution) is to be applied, at his request, to the purchase of a horse ambulence for the conveyance of injured persons to the hospital, a rearrangement of the heating and ventilating, and other much-needed improvements.

THE Duke of Connaught speaking at the annual meeting of the Royal Portsmouth Hospital, stated thatthe balance-sheet showed a deficit of £1,000 and though there might be a difference of opinion as to the management—the hospital was a splendid institution, and the medical work was carried out admirably.

THE total number of in-patients to the Sussex County Hospital during 1891 was 1,532. The average period of each patient in the hospital was 38·6 days. Total number of out-patients 10,430. A "Special Building Fund" has been opened for the purpose of providing additional accommodation for women.

THE old east wing of the Longmore Hospital for Incurables, Edinburgh, has been pulled down, and another wing is being built with accommodation for 34 additional patients. When the alterations are completed there will be accommodation for about 100 patients. The report states that the hospital is in need of subscribers.

THE annual statement of accounts of the Bedford Provident Dispensary shows that the institution is steadily increasing. The provident members' subscriptions (£848 14s. 8d.) were again larger than in any previous year. The Surgeons' Fund, with a total of £1,317 3s. 5d., shows a balance of £412 16s. 8d. The balance at the bank is £499 13s. 4d.

THE legacies received by the Middlesex Hospital during the past year amounted to £9,911 and included a further benefaction from the estate of the late Sir Joseph de Courcy Laffan of £3,526 to the Cancer Fund. In 1891, 41,336 in and out patients were treated, the average number of beds occupied daily being 254. The expenditure exceeded the income by £12,027.

FROM May 1st to December 12th of last year 587 patients were admitted to the Royal Northern Sea-Bathing Infirmary, Scarborough. The Committee have decided to make some additions at the north end of the infirmary, which will afford increased accommodation required for the staff on the first floor, and provide another ward of eight beds for patients.

LORD CARRINGTON, presiding in the Hotel Metropole over the festival dinner in support of the East London Hospital for Children, stated that it was not endowed, it had afforded relief to 16,500 in-patients, and its yearly expenditure was £7,000, whereas the total income from invested funds was only £305. By the festival this excellent charity will benefit to the extent of £1,960.

AT the annual meeting of the managers of the Hospital Saturday Fund held on Saturday, the 20th inst., at the Mansion House, the Lord Mayor presiding, it was reported that the receipts for the year had been £19,683, against £20,333 in 1890, and the expenses £2,819, as against £2,961 in 1890. The number of institutions participating in the fund was 150, as against 144 in 1890.

THE annual report of the Kent and Canterbury Hospital states that the institution is free from debt and starts the year with a good balance in hand. During the past year 687 in-patients (as against 747 in 1890), 1,220 out-patients (as against 1,628 in 1890), were treated. The receipts were £6,194 9s. 5d., exceeding those of 1890 by £518 14s. 8d., while the expenditure was £526 15s. less.

A STRANGE case of apparent death reaches us from St. Petersburg. A lady who had been suffering from a violent nervous attack, followed by syncope, after a time ceased apparently to breathe. Upon the microphone being applied a faint sound was detected, which proved that she was still alive. Restoratives were applied, and she recovered. The incident suggests a new safeguard against premature burial or cremation.

AT the 41st annual meeting of the Governors of the Cancer Hospital (Free), Brompton, Sir George Samuel Measom, J.P., presiding, the report showed that during the past year 1788 new patients were received, and the total number of attendances of out-patients was 7,498. An earnest appeal for continued support was made by the Committee. The reliable income is about £3,000 a-year less than the ordinary expenditure.

### THE STUDENT OF MEDICINE.

[All communications for this Supplement should be addressed to THE EDITOR of THE HOSPITAL, at 140, Strand, with the words, "Students' Column," written in the left hand top corner of the envelope.]

### APPOINTMENTS.

T. C. Allbutt, M.D., LL.D., F.R.S., appointed Regius Professor of Physic in the University of Cambridge. P. J. Atkey, L.R.C.P., M.R.C.S., appointed Assistant House Surgeon to St. Thomas's Hospital. A. Banks, L.R.C.P., M.R.C.S., appointed Senior Obstetric House Physician to St. Thomas's Hospital. T. H. Bishop, M.R.C.S.Edin., appointed House Surgeon to the Royal Portsmouth Hospital. C. K. Bond, L.R.C.P.Lond., M.R.C.S., D.P.H., appointed Medical Superintendent of the Bradford Corporation Fever Hospital. C. R. Box, B.Sc.Lond., L.R.C.P., M.R.C.S., appointed House Surgeon to St. Thomas's Hospital. H. Burden, L.R.C.P., M.R.C.S., appointed Assistant House Surgeon to St. Thomas's Hospital. A.W. Cadman, L.R.C.P.I., M.R.C.S.Eng., appointed Demonstrator of Anatomy at King's College. W. H. Dolamore, L.R.C.P.Lond., M.R.C.S., L.D.S., appointed Medical Tutor to the London School of Dental Surgery. F. R. Fairbank, M.D.Heid., &c., appointed Consulting Surgeon to the Doncaster General Infirmary and Dispensary. T. A. M. Ford, L.R.C.P., M.R.C.S., appointed House Surgeon to St. Thomas's Hospital. T. H. Haydon, B.A., M.B., B.C.Cantab., L.R.C.P., M.R.C.S., appointed Junior Obstetric House Physician to St. Thomas's Hospital. T. H. Kellock, M.A., M.B., B.C.Cantab., F.R.C.S., L.R.C.P., appointed House Surgeon to St. Thomas's Hospital. C. Latter, B.A., M.B., B.C.Cantab., appointed Resident House Physician to St. Thomas's Hospital (extension). H. A. Lownds, L.R.C.P. and S.Edin., appointed Surgeon to the Doncaster General Infirmary and Dispensary.

### VACANCIES.

#### Resident Medical Officers.

Birmingham and Midland Eye Hospital, Assistant House Surgeon—Salary £50, &c.

Bridgwater Infirmary, House Surgeon—Salary £80, &c.,

Camberwell House Asylum, Camberwell, S.E., Junior Medical Officer; unmarried—Salary £100, &c.

Central London Ophthalmic Hospital, 328A, Gray's Inn Road, W.C., House Surgeon.

County Asylum, Rainhill, near Liverpool, Assistant Medical Officer; unmarried, and not more than 30 years of age—Salary £100, &c.

Denbighshire Infirmary, Denbigh, House Surgeon. Must be conversant with the Welsh language—Salary £85, &c.

Hoxton House Asylum, N., Assistant Medical Officer—Salary £120, &c. rising £10 a-year to £160.

Leeds Public Dispensary, Junior Resident Medical Officer—Salary £85,

Lincon County Hospital, House Surgeon; doubly qualified; unmarried, and under 40 years of age—Salary £100, &c.

Manchester Royal Infirmary, Assistant Medical Officer for the Monsall Fever Hospital—Salary £100, &c.

Montrose Royal Lunatic Asylum, Junior Assistant Medical Officer—Salary £80, &c.

National Hospital for the Paralysed and Epileptic, Queen's Square, Bloomsbury, Senior House Physician; doubly qualified—Salary £100, &c.

Norfolk County Asylum, Thorpe, Norwich, Med'c l Officer, single, and under 30 years of age; appointment for five years—Salary £160, &c.

North-Eastern Hospital for Children, Hackney Road, N.E., Junior House Surgeon; doubly qualified—Salary at the rate of £60, &c.

Paddington Union, W., Assistant to the Medical Superintendent of the Infirmary, and Assistant Medical Officer of the Workhouse: doubly qualified; unmarried—Salary £100, rising £5 annually to £120, &c.

Rotherham Hospital, Resident House Surgeon, doubly qualified, unmarried—Salary £100, &c.

West London Hospital, Hammersmith Road, W., House Physician.

West London Hospital, Hammersmith Road, W., House Surgeon.

Weston-Super-Mare Hospital, House Surgeon; unmarried; doubly qualified—Salary £50, &c.

#### Non-Resident Medical Officers.

Cancer Hospital, Free, Brompton, S.W., two Surgeons; must be F.R.C.S.Eng., and reside within the four miles radius.
Dental Hospital of London, Leicester Square, two Assistant Dental Surgeons; must be Licentiates in Dental Surgery.
Dental Hospital of London and London School of Dental Surgery, Leicester Square, Demonstrator—Honorarium, £50.
London Lock Hospital and Asylum, Harrow Road, W., and 91, Dean Street, Soho, W.—Registrar.
London Temperance Hospital, Hampstead Road, N.W., Physician, doubly qualified.
London Temperance Hospital, Hampstead Road, N.W., Assistant Physician, doubly qualified.
Metropolitan Hospital, Kingsland Road, N.E., two Assistant Surgeons must be F.R.C.S.Eng.

